# 

**Published:** 1891-10

**Authors:** 


### Society Meetings.

The District Medical Societies of Northern Ohio, comprising the North-Western Ohio Medical Society, the North Central Ohio Medical Society, and the North-Eastern Ohio Medical Society, will hold a joint meeting at Mansfield, on Thursday, Friday, and Saturday, November 5th, 6th, and 7th. On Thursday evening, the members will be entertained by a reception given in honor of the association by the Hon. John Sherman, and on Friday evening by a reception given by the Hon. M. D. Harter. Every arrangement has been made to make this meeting a pleasant and profitable one, and we trust that a full attendance may be had. Ample hotel accommodations will be arranged for; and an effort will be made to secure reduced rates on all the railroads of Ohio. The following committee of arrangements has been appointed: Dr. R. Harvey Reed, Dr. J. W. Craig, and Dr. Geo. Mitchell, all of Mansfield.

JAMES A. DUNCAN, M. D.,
X. C. SCOTT, M. D.,
R. HARVEY REED, M. D.,
*Joint Committee.*

New York State Society of Railway Surgeons.—The first meeting of this Society will be held in the parlors of the Hotel Bensonhurst, at Bensonhurst-by-the-Sea, Long Island, October 27,

1891. A full attendance is desired ; programmes of the meeting may be obtained by addressing Dr. Geo. Chaffee, 201 47th street, Brooklyn.

### BOOKS AND PAMPHLETS RECEIVED.

Regional Anatomy in its Relation to Medicine and Surgery. By George McClellan, M. D., Lecturer on Descriptive and Regional Anatomy at the Pennsylvania School of Anatomy; Professor of Anatomy at the Pennsylvania Academy of the Fine Arts; member of the Association of American Anatomists, Academy of Natural Sciences, Academy of Surgery, College of Physicians, etc., of Philadelphia. Illustrated from photographs taken by the author, of his own dissections, expressly designed and prepared for this work, and colored by him after nature. "L'anatomie n'est pas telle qu'on l'enseigne dans les ecoles."—Bichat. In two volumes. Vol. I. Philadelphia: J. B. Lippincott Company. 1891.

Tables for Doctor and Druggist. Comprising: I. Table of Solubilities. II. Table of Reactions and Incompatibles. III. Table of Doses and Use of Medicines. IV. Table of Specific Gravities. V. Table of Poisons and Antidotes. Compiled by Eli H. Long, M. D, Professor of Materia Medica Buffalo College of Pharmacy; Adjunct Professor of Materia Medica Medical Department, University of Buffalo. Detroit: Geo. S. Davis, Medical Publisher. 1891.

Pulmonary Consumption, a Nervous Disease: Considered as such from a Practical, a Clinical, and Therapeutic Standpoint. By Thomas J. Mays, M. D., Professor of Diseases of the Chest in the Philadelphia Polyclinic and College for Graduates in Medicine; Visiting Physician to the Rush Hospital for Consumption of Philadelphia; Fellow of the College of Physicians of Philadelphia; member ot the Philadelphia County Medical Society; member of the Neurological Society of Philadelphia, etc., etc. Physicians' Leisure Library. Geo. S. Davis, Detroit, Mich. 1891. Price, cloth, 50c.; paper, 25c.

Essentials of Physiology arranged in the form of Questions and Answers, prepared especially for students of medicine. By H. A. Hare, B. Sc., M. D., Professor of Therapeutics and Materia Medica in the Jefferson Medical College of Philadelphia; Physician to St. Agnes's Hospital, and to the Medical Dispensary of the Children's Hospital; Laureate of the Royal Academy of Medicine in Belgium, of the Medical Society of London, etc.; Secretary of the convention for the revision of the Pharmacopeia. 1890. Saunders' Question-Compends, No. 1. Third edition, thoroughly revised and enlarged, by the addition of a series of handsome plate illustrations taken from the celebrated Icones Nervorum Capitis of Arnold. Philadelphia: W. B. Saunders, 913 Walnut street. 1891.

Addresses, Papers, and Discussions in the Section of Obstetrics and Diseases of Women at the Forty-second Annual Meeting of the American Medical Association, at Washington, D. C., May 5-8, 1891. Chicago: Printed at the office of the Association. 1891.

A Short Manual of Analytical Chemistry, Qualitative and Quantitative, Inorganic and Organic. Arranged on the principle of the course of instruction given at the South London School of Pharmacy. By John Muter, M. A., Ph. D., F. R. S. E., F. I. C., F. C. S., Analyst to the Metropolitan Asylum Board; Public Analyst for Lambeth, Wansworth, South wark, Newington, Rothershithe, and the Linsey Division of Lincolnshire; Past President of the Society of Public Analysis; author of Pharmaceutical and Medical Chemistry (Theoretical and Descriptive), A Key to Organic Materia Medica, late editor of *The Analyst*, etc., etc. First American from the fourth English edition, edited by Claude C. Hamilton, M. D., Ph. G., Professor of Analytical Chemistry in the University Medical College and the Kansas City College of Pharmacy; Professor of Chemistry in the Western Dental College of Kansas City, Missouri. Philadelphia: P. Blakiston, Son & Co., 1012 Walnut street. 1891.

Essentials of Anatomy and Manual of Practical Dissection, together with the Anatomy of the Viscera. Prepared especially for Students of Medicine. By Charles B. Nancrede, M. D., Professor of Surgery and of Clinical Surgery in the University of Michigan, Ann Arbor; Corresponding member of the Royal Academy of Medicine, Rome, Italy; late Senior Surgeon to Episcopal Hospital; late Surgeon to Jefferson Medical College Hospital; formerly Lecturer on Osteology, etc., in Medical Department, University of Pennsylvania; late Professor of General Orthopedic Surgery in Philadelphia Polyclinic, etc. Fourth edition, revised and enlarged by an Appendix containing Hints on Dissection, by J. Chalmers Da Costa, M. D., based upon the last edition of Gray's Anatomy. Thirty handsome full-page lithographic plates in colors, and 188 fine wood cuts. Philadelphia: W. B. Saunders, 913 Walnut street. 1891.

Therapeutics: Its Principles and Practice. By H. C. Wood, M. D., LL. D., Professor of Materia Medica and Therapeutics, and Clinical Professor of Diseases of the Nervous System, in the University of Pennsylvania. A Work of Medical Agencies, Drugs and Poisons, with Especial Reference to the Relations between Physiology and Clinical Medicine. The eighth edition of Treatise on Therapeutics, re-arranged, re-written, and enlarged. Philadelphia: J. B. Lippincott Company. 1891.

Syllabus of the Obstetrical Lectures in the Medical Department of the University of Pennsylvania. By Richard C. Norris, A. M., M. D., Demonstrator of Obstetrics, University of Pennsylvania; Physician to the Methodist Episcopal Hospital; Obstetrical Registrar, Philadelphia Hospital. Second edition. Philadelphia: W. B. Saunders. 1881.

Second Report of the Johns Hopkins Hospital. For the Year ending January 31, 1891. Baltimore: The Johns Hopkins Press. 1891.

Sulphuring or Bleaching Dried Fruit a Mistake, If Not a Crime. By Joel W. Smith, M. D., Charles City, Iowa.

Conditions Following Parturition Requiring Abdominal Section. By E E. Montgomery, M. D., Philadelphia. Reprint: Transactions Med. Soc. State of Penn. 1891.

Three Cases of Epithelial Grafting from the Horny Epidermis, with remarks by C. R. Kibler, M. D., of Corry, Pa. Reprinted from the *Journal of the American Medical Association*, August 8, 1891.

Evidence of Arsenical Poisoning in the Snook-Herr Wedding Guests. By J. W. Irwin, M. D. Reprint: *New York Medical Journal*, August 1, 1891.

Addresses, Papers, and Discussions in the Section of Surgery and Anatomy, at the Forty-second annual meeting of the American Medical Association, at Washington, D. C., May 5–8, 1891. Chicago: Printed at the office of the Association. 1891.

Therapeutics of Diphtheria. By Dr. Joseph Burghardt, Practising Physician in Vienna. From a lecture delivered at the meeting of the Council of Vienna Physicians, April 8, 1889. Translated by Charles Raettig. Translation authorized by the Lecturer. New York: J. H. Vail & Co. 1891.

One Thousand Cases of Labor, and Their Lessons. By G. W. H. Ex-Kemper, M. D., Member of the Delaware County (Ind.) Medical Society; President of the Indiana State Medical Society, Muncie, Ind. Reprint: *The Medical News*, Sept. 12, 1891.

On Unusual Methods of Acquiring Syphilis, with Reports of Cases. By L. Duncan Bulkley, A. M., M. D., of New York. Reprint: *The Medical News*, March 2–9, 1889.

Proceedings and Addresses at a Sanitary Convention held at Niles, Mich., Feb. 5 and 6, 1891, under the direction of the State Board of Health and a committee of citizens of Niles. Lansing: Robert Smith & Co., State Printers and Binders. 1891.

A Few Thoughts on the Technique of Hysterectomy. By Joseph Eastman, M. D., President and Professor of Diseases of Women and Abdominal Surgery, Central College of Physicians and Surgeons, Indianapolis. Reprint: *The Medical News*, August 1, 1891.

A Clinical Study on Alopecia Areata and its Treatment. By L. Duncan Bulkley, A. M., M. D., New York. Reprint: *The Medical News*, March 2, 1889.

On the Value of Frequently-Repeated Doses of Arsenic in the Treatment of Bullous Diseases of the Skin, especially in Children. By L. Duncan Bulkley, A. M., M. D. New York. Reprint: *The New York Medical Journal* for April 13, 1889.

Cornell University Agricultural Experiment Station. Bulletin 29. Agricultural and Chemical Divisions. July, 1891. Cream Raising by Dilution. II. The Effect of a Delay in Setting on the Efficiency of Creaming. III. Application of Dr. Babcock's Centrifugal Method, to the Analysis of Milk, Skim-Milk, Buttermilk, and Butter. IV. The Relation of Fibrin to the Effectual Creaming of Milk. Published by the University, Ithaca, N. Y. 1891.

---

NOTICE TO CONTRIBUTORS. — We are glad to receive contributions from every one who kows anything of interest to the profession. Articles designed for publication in the JOURNAL should be handed in before the first day of the month. The Editors are not responsible for the views or opinions of contributors. All communications should be addressed to the Managing Editor, 284 FRANKLIN STREET, BUFFALO, N. Y.

